# 3D-exoscopic microlaryngoscopy in phonosurgery for glottic insufficiency

**DOI:** 10.1007/s00405-023-08345-7

**Published:** 2023-12-18

**Authors:** Hans N. C. Eckel, Antonia Nolte, Martin S. Otte, Sami Shabli, Ruth Lang-Roth, Jens P. Klußmann, Kevin K. Hansen

**Affiliations:** Department of Otorhinolaryngology, Head and Neck Surgery, Medical Faculty, University of Cologne, Kerpener Str. 62, 50931 Cologne, Germany

**Keywords:** Injection laryngoplasty, 3D-exoscope, Vocal fold augmentation, Vocal fold paralysis, Voice

## Abstract

**Purpose:**

We hypothesized that using a 3D-exoscope (3Dex) in microlaryngoscopic phonosurgery is non-inferior to using a standard operating microscope (OM). To compare the above, we utilized a 3Dex and an OM for microlaryngoscopic vocal fold augmentation with autologous fat in patients with glottic insufficiency and compared the procedure itself and the long-term impact of vocal fold augmentation on subjective and objective voice parameters in both groups.

**Methods:**

36 patients with glottic insufficiency received microlaryngoscopic laryngeal augmentation with autologous fat. A 3Dex was utilized in 24 cases for visualization and compared to twelve cases in which an OM was used. Voice parameters were evaluated over a period of twelve months.

**Results:**

Comparison of operation time and voice parameters between the 3Dex and OM groups did not reveal significant differences. Significant improvement of mean voice quality in all parameters excluding roughness was observed at 3 and 6 months followed then by a slight decrease of voice quality parameters between the 6 and 12 months interval in both groups.

**Conclusion:**

Our findings indicate no difference concerning operation time and outcome between the use of a 3Dex and an OM in phonosurgery. Our results highlight a significant voice improvement after vocal fold augmentation with autologous fat in glottic insufficiency mediated dysphonia. The smaller viewing system, better ergonomics for the primary surgeon and the assistant and a direct view for the entire surgical team make a 3Dex an interesting alternative for visualization in microlaryngoscopic phonosurgery.

## Introduction

Glottic insufficiency is a condition of the larynx characterized by incomplete closure of the vocal folds causing inappropriate leakage of air through the glottis on attempting to phonate. This can lead to a significant reduction in the quality of life of affected patients due to chronic dysphonia and altered efficacy of swallowing and coughing, leading to an increased risk of aspiration [1]. Unilateral vocal fold paresis (UVFP), age related changes of the larynx and defects in the vocal fold mucosa following surgical interventions are frequent causes of glottic insufficiency and hence dysphonia [2–5]. The origin of vocal fold paresis may be iatrogenic, idiopathic, or caused by malignant tumors or trauma. Glottic insufficiency, caused by a vocal fold soft tissue defect, can be the consequence of an ablative surgery for benign or malignant vocal fold lesions, as well as due to neurodegenerative disease such as Parkinson [5–8].

Voice rehabilitation is a common treatment option for glottic insufficiency. If the outcome of rehabilitation is not satisfactory, surgical interventions may be applied to decrease the glottic gap, thus restoring glottic competence. This can be achieved either by medialization of the vocal fold through an external transcervical approach using an implant (thyroplasty), transoral vocal fold augmentation (VFA) or laryngeal reinnervation in UVFP [3, 9–12]. Materials commonly used for augmentation include temporary fillers such as hyaluronic acid and permanent materials including Calcium Hydroxylapatite (RADIESSE®) and Polyacrylamide (Aquamid®), however local and systemic foreign body reactions can occur [13]. Alternatively, autologous fat may be used [14–16]. By many authors, fat is not considered a permanent material due to potential resorption, but others have reported long-term improvement of voice quality following augmentation with autologous fat hinting at a potentially permanent effect [10, 17–20].

In microlaryngoscopy, the established method of visualization is the operating microscope (OM). Beginning in 2019, we have implemented the use of a 3D-Exoscope (3Dex) system in microlaryngoscopic phonosurgery, aiming to further advance this surgical procedure by improving visualization of the glottis. Applied first in neurosurgery [21], urology [22], and gynecologic surgery [23], the use of 3Dex systems is now growing in ENT surgery as well [24–27]. At present, only small series of using an 3Dex in microlaryngoscopy have been reported in the literature and to the authors' knowledge, no comparative reports exist on the application of a 3Dex in microlaryngoscopic phonosurgery.

This article aims to compare the use of a 3Dex with the use of an OM by describing the application of the above in the microlaryngoscopic procedure of autologous fat injection in patients with glottic insufficiency due to UVFP. We chose vocal cord augmentation as an index procedure because it is a standardized procedure with few potential setbacks when compared to TOLS, in which tumor size, surgeon experience, amount of bleeding and quality of laryngeal exposure may greatly influence variables such as operation time. Furthermore, voice quality development over time is a variable that is well suited to evaluating the impact of surgery during follow up. Particular attention is placed on the feasibility of the 3Dex system compared to the established surgical microscope in microlaryngeal phonosurgery. Secondary endpoints are the long-term effect of autologous fat transplantation for glottic insufficiency.

## Methods

### Patients

A total of thirty-six consecutive patients were treated with unilateral or bilateral autologous fat vocal fold augmentation between March 2019 and December 2022 at the department of oto-, rhino-, laryngology, head and neck surgery of the University Hospital of Cologne. Patients aged ≥ 18 years with dysphonia due to glottic insufficiency were Included and follow-up was conducted for the duration of one year. All patients had undergone voice rehabilitation prior to surgery. Ascertained patient information included demographics, surgical data, diagnosis and clinical follow-up. Videostroboscopy was performed in all patients using the XION (Berlin, Germany) video laryngoscopy system.

Diagnostic evaluation by videostroboscopy and voice quality was performed preoperatively as well as three, six and twelve months after surgery. Voice quality was evaluated by subjective and objective parameters. Objective data were collected using maximum phonation time (MPT) and the RBH scale. This scale includes three domains (hoarseness, breathiness, and roughness) which are graded from 0 to 3 by two voice professionals. A score of 0 indicates a healthy voice while a score of 3 marks severely impaired voice quality. Patients’ subjective voice quality was assessed using the Voice Handicap Index 30 (VHI30) questionnaire, a self-assessment with 3 subscales that measure the emotional and functional aspects of voice impairment. Scores range from 0 to 120 and a higher score indicates a higher degree of voice deterioration.

### Vocal fold augmentation procedure

**Fig. 1:**
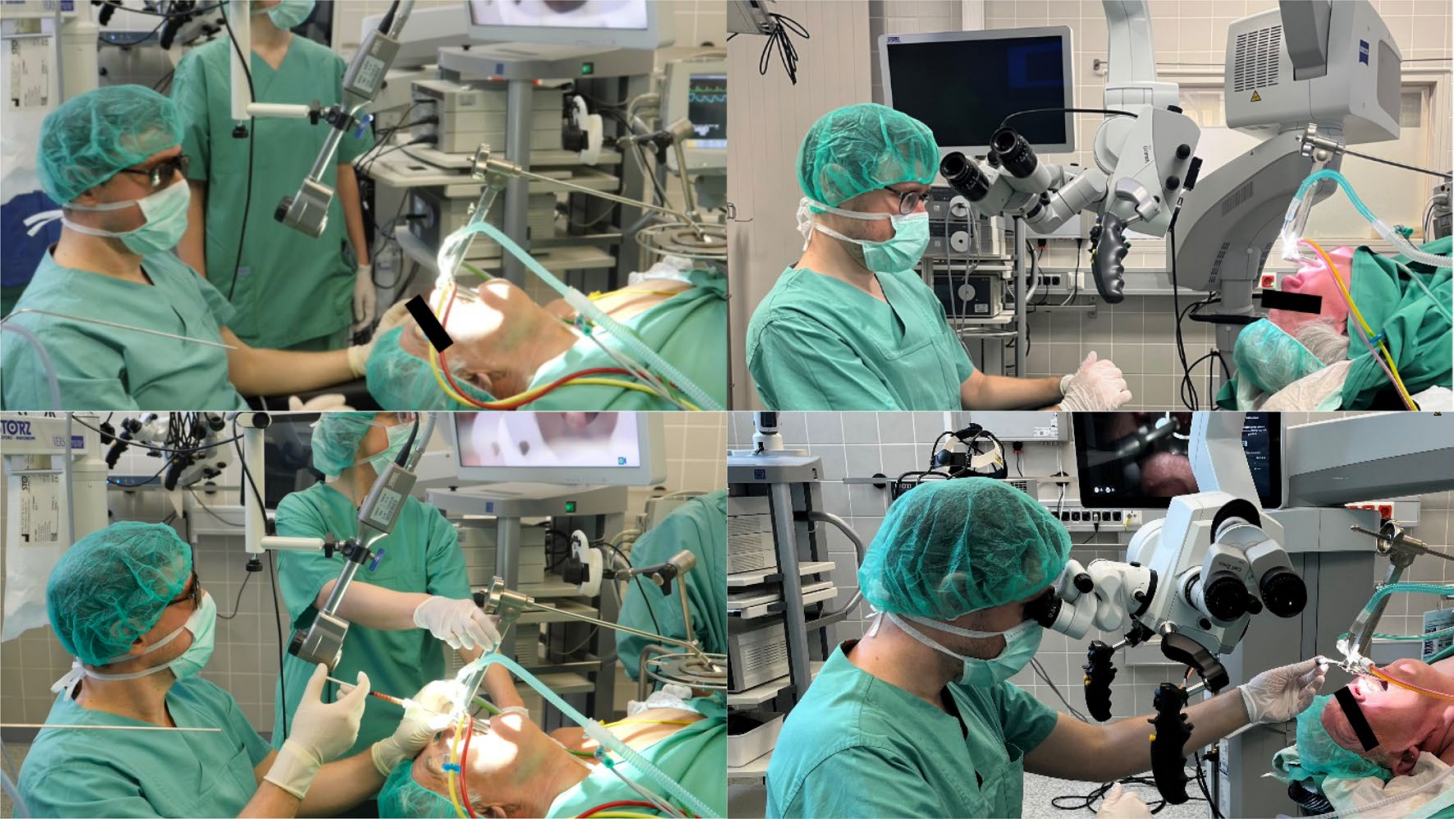
Intraoperative pictures of transoral laryngeal surgery using the 3Dex (left panels) or a conventional operating microscope (right panels). The smaller optical system allows for easier maneuverability of surgical instruments and improved surgeon ergonomics in microlaryngoscopy; 3Dex, 3D exoscope

The procedure was performed under general anesthesia. First, fatty tissue was harvested from the lower abdomen using a 2mm diameter liposuction canula for fat harvesting (VoiceInject, Spiggle & Theiss). The collected fatty tissue was centrifuged at 3000 rounds per minute for the duration of 3 min to separate fat cells from liquid fat and debris. Fat cells were transferred to 1ml Luer-Lock syringes to be used for augmentation. For visualization of the glottic space during microlaryngoscopy, either a conventional operating microscope (Tivato 700, Carl Zeiss) or the 3Dex were used. The intraoperative microlaryngoscopy setup for both visualization methods is displayed in Fig. 1.

The 3Dex (VITOM, Karl Storz) consists of a high-definition or 4K video camera with optical and digital zoom and a fiber optic-delivered or L.E.D. light source [1, 2]. This system is suspended above the surgical field with a manually actuated articulating holder or robotic arm, which transmits a two-dimensional (2D) or 3D image to a high-resolution monitor placed at eye level directly across from the surgeon. Magnification power of 8–30 is possible through the 3D camera, while the depth of field ranges from 7 to 44 mm, with a focal distance of 20–50 cm, allowing the surgical field to be observed and illuminated at various distances from the patient [28].

Fat injection was performed by injection into the paraglottic space with a 0,9 mm diameter injection needle (VoiceInject, Spiggle & Theiss). Injection volumes ranged from 1 ml to 2,5 ml, the cannula was left in place after injection for one minute to diminish fat extrusion through the injection canal. More than two injection sites were avoided to prevent fat extrusion from previous injection sites. In case of UVFP and age-related vocal fold atrophy, the injection was performed laterally of the vocal muscle into the paraglottic space. Operations were performed by J.P.K. and K.H. (authors of this manuscript) at the University Hospital of Cologne.

### Statistical analysis

Data was collected retrospectively from patients’ medical records and entered in an Excel 2016 (Microsoft, Redmond, WA) spreadsheet. Statistical analysis was performed using Graph pad Prism Version 8.3. (GraphPad Software, San Diego, CA). Paired *t* test was used to compare the data before and after surgery. Two-sided exact tests were used and *P*-values < 0.05 were considered significant. *P*-values of statistical significance were marked with asterisks as indicated in figure legends. Patient characteristics were compared using students *T* test, Fisher’s exact test and two-way ANOVA where applicable. The amount of improvement in voice parameters (Δvoice parameter) after surgery, using either a conventional operating microscope or the 3Dex, were compared between the two groups using the Mann–Whitney *U*-test. A *P*-value < 0.05 was considered as statistically significant.

## Results

### Patient characteristics

Thirty-six patients with a mean age of 58 years (range, 20–85 years) were treated for dysphonia by vocal fold augmentation, mean age was comparable in both groups (Table 1). Unilateral Recurrent nerve paresis, caused by extra-laryngeal malignant tumors, systemic disease, iatrogenic or idiopathic origin, was the most common diagnosis causing dysphonia in patients who received VFA with autologous fat. UVFP was diagnosed in 24/36 (66.6%) patients. Distribution of cause for UVFP was comparable in both groups (Table 1). Ten patients (28%), had dysphonia due to previously treated laryngeal cancer and two patients received VFA due to age-related deterioration of the larynx (presbyphonia). Three patients had undergone VFA with hyaluronic acid at least three months before receiving an injection with autologous fat and three patients reported previous VFA with a permanent filler (Calcium Hydroxylapatite) without satisfactory improvement of voice quality. One patient received arytenoid cartilage medialization surgery more than 20 years before VFA. In the 3Dex cohort, 4/24 (17%) patients received bilateral VFA while 3/12 (25%) patients in OM group received bilateral injection. All other patients received unilateral VFA. The mean duration of surgery was 32 ± 9.798 (mean ± standard deviation) minutes in the 3Dex group and 34 ± 8.185 min in the OM group (Table 1). Injection volume did not differ significantly between the3Dex and the OM group (1.6 ± 0.5477mL and 1.425 ± 0.5029mL respectively, *P* = 0.3699). Importantly, the difference in duration of surgery was not statistically significant (*P* = 0.7919) when comparing 3Dex and OM groups, demonstrating that 3Dex assisted surgery did not increase the duration of the procedure (Table 1).

**Table 1:** Patient characteristics, diagnosis, and surgical parameters of 36 included patients given as mean ± standard deviation. Age, Duration of surgery and Injected volume given as mean ± standard deviation. Abbreviations: UVFP, unilateral vocal fold paresis

	3D Exoscope (*N* = 24)	Microscope (*N* = 12)	*P*-value
Patient characteristics			
Age (years)	58 ± 12.83	58.67 ± 21.62	0.7219
Sex (female: male)	10: 14	04: 08	0.2890
Diagnosis			
Iatrogenic/disease related UVFP	12 (50%)	4 (33.3%)	0.1817
Idiopathic UVFP	5 (20.8%)	3 (25%)	
Presbyphonia	1 (4.2%)	1 (8.4%)	
Previous Laryngeal cancer	6 (25%)	4 (33.3%)	
Surgical parameter			
Duration of surgery (Minutes)	33.21 ± 9.798	34.08 ± 8.185	0.7919
Injected volume (mL)	1.6 ± 0.5477	1.425 ± 0.5029	0.3699

### Vocal fold augmentation improved voice quality

All patients completed preoperative voice evaluation including MPT, VHI 30 questionnaire and RBH scale. Patients were asked to attend follow-up assessment of voice quality 3, 6 and 12 months after surgery. Follow-up analyses were attended by 28 (78%), 21 (58%) and 19 (53%) patients after three, six and twelve months respectively.

**Fig. 2:**
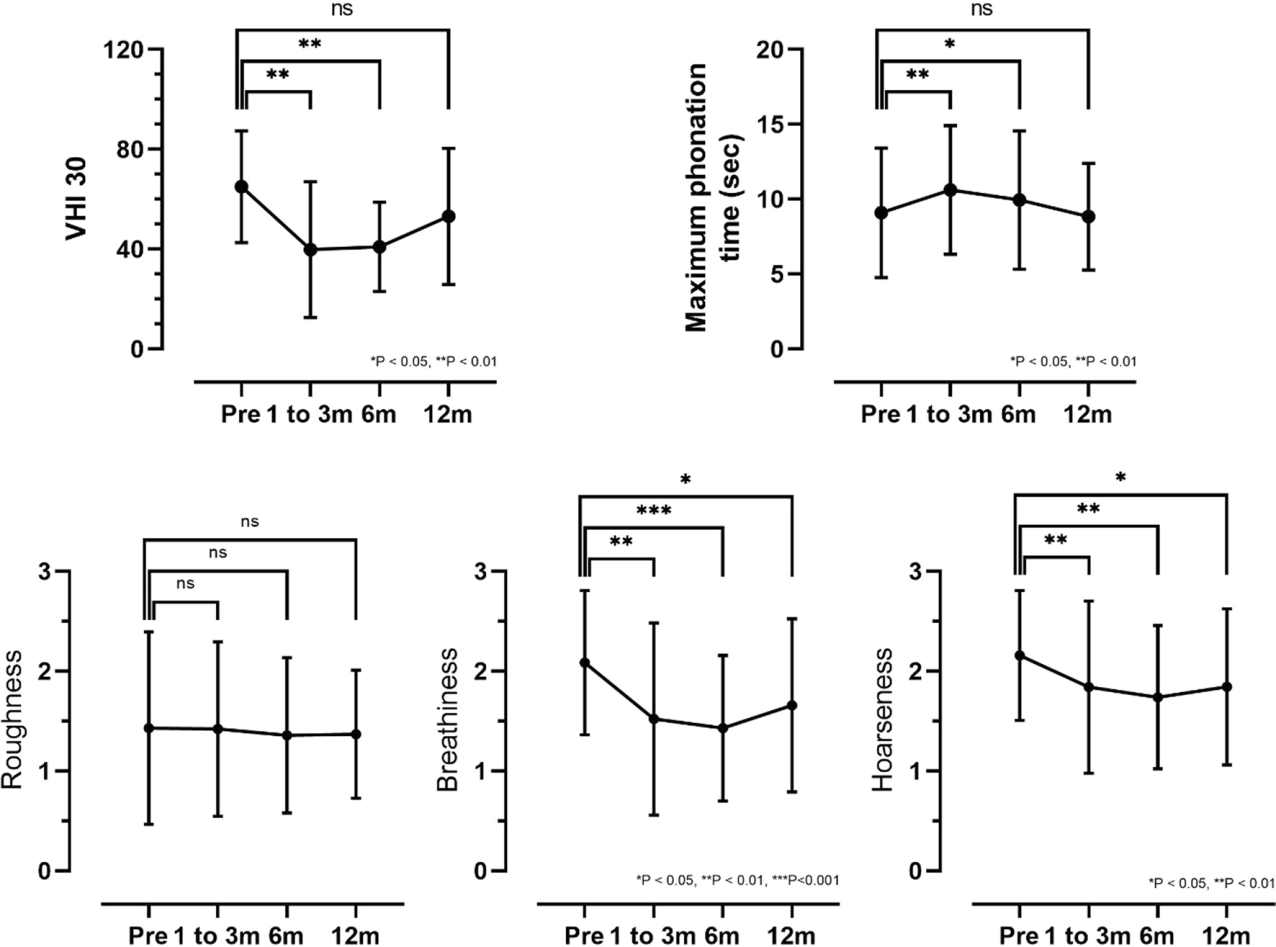
VHI 30, Maximum phonation time, Roughness, Breathiness, Hoarseness for n = 36 included patients before surgery and at follow up examinations 1 to 3, 6 and 12 months after surgery. Abbreviations: VHI 30, Voice Handicap Index 30; ns, not significant; sec, seconds; m, months

Temporary improvement of subjective voice quality, as measured by the VHI 30 questionnaire, was reported 3 and 6 months after VFA (*P* < 0.001) (Fig. 2). While a trend of lower median VHI score from 65 ± 22.41 preoperatively to 53.1 ± 27.3 12 months after surgery remained, this improvement did not reach statistical significance (*P* = 0.372). Data lost to clinical follow up was comparable in both groups.

External voice evaluation by two specialized voice therapists showed significantly improved hoarseness and breathiness of voice at all timepoints after surgery (Fig. 2). Roughness of the voice remained at a similar level after VFA in comparison to preoperative assessment. Maximum phonation time was significantly improved at three- and six-month controls, but did not remain significantly increased after twelve months. Voice specialists were not aware of patient being in 3Dex or OM group, the same tests were applied for follow up in both groups.

### 3Dex—or operating microscope assisted surgery did not impact postoperative voice improvement

**Fig. 3:**
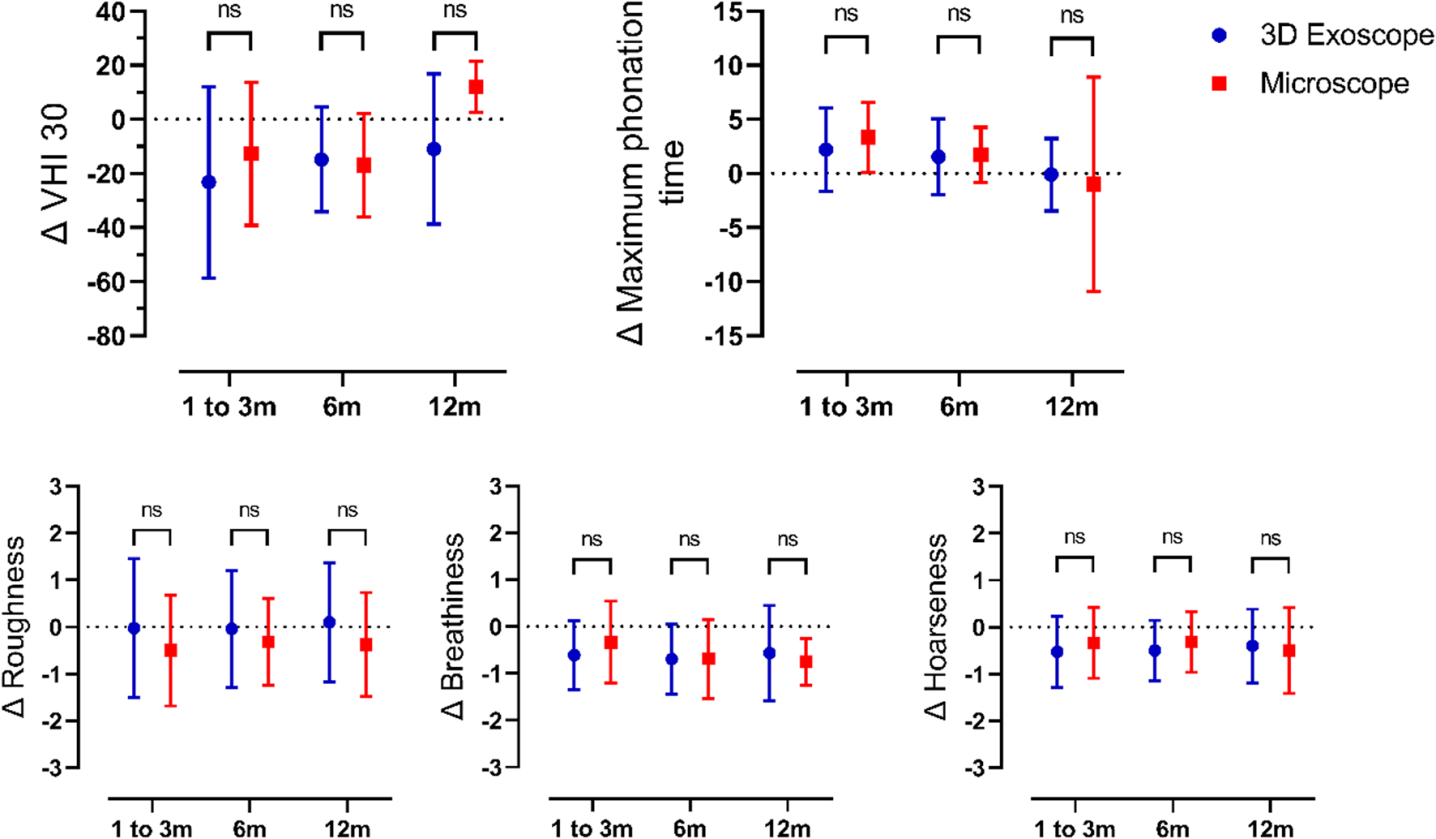
ΔVHI 30, Δ Maximum phonation time, Δ Roughness, Δ Breathiness, Δ Hoarseness compared to preoperative evaluation for patients at follow up examinations 3, 6 and twelve months after surgery for 3Dex (blue) and Microscope (red). Abbreviations: VHI 30, Voice Handicap Index 30; ns, not significant; sec, seconds; m, months;

We calculated the amounts of voice improvement Δ (difference between before and after surgery at given point in time) after VFA compared to preoperative evaluation of objective and subjective voice parameters. To elucidate whether visualization of the glottis during microlaryngoscopy for VFA with a conventional OM or the 3Dex had a significant impact on voice quality, we compared the improvement of voice parameters between the two patient cohorts. Self-reported improvement of voice quality, quantified by a reduced VHI 30 score, was not significantly higher in the OM group compared to patients augmented using the 3Dex (Fig. 3). Likewise, improvement of MPT, breathiness and hoarseness did not show marked differences between the two groups (Fig. 3). Voice roughness did not improve independent of visualization of the glottis with the 3Dex or OM.

## Discussion

Recent literature highlights an increasing interest in testing the feasibility of exoscopic systems for replacement of the operating microscope in transoral head and neck procedures [29, 30]. Advantages reported include a smaller size of the viewing system, resulting in better ergonomics for the primary surgeon and the assistant, providing a direct view for the entire surgical team, improving the efficacy of the assistant’s maneuvers (four hands surgery), and permitting a high quality for teaching purposes in the operating theater (Fig. 1) [28].

Recordings of entire surgical procedures in 3D-HD are also easily realized as previously demonstrated by our team [31]. Vocal fold augmentation is a well-established and routinely applied phonosurgical procedure for patients with persistent dysphonia due to decreased glottic competence. It is the impression of the authors that the increased depth of field offered by the 3Dex when compared to the OM helps with optimal needle placement during this procedure, concerning injection depth in laryngeal augmentation procedures and thus facilitates correct placement of fat tissue on the level of the glottic plain, therefore avoiding initial injection into the subglottic space and the necessity of injection depth correction. This opinion was however not backed up, as our data shows no significant differences in voice outcome between both groups. This may be due to additional injection into the subglottic space not impacting voice improvement as long as the vocal fold is sufficiently medialized. No significant differences were observed concerning duration of operation or voice outcomes between OM and 3Dex groups thus demonstrating non-inferiority of the use of an 3Dex compared to the OM for laryngeal augmentation procedures.

The secondary endpoint of the study was long term voice result after VFI with autologous fat. An ongoing discussion exists in the literature concerning the classification of autologous fat as permanent vs. semi-permanent material for augmentation procedures. While some authors describe a permanent effect of autologous fat and associate this with the application of specific injection techniques[17] or the effect of stem cells and growth factors [32] progressive fat resorption over time is described elsewhere [33, 34]. Some authors go as far as stating fat injection merely as a precursor procedure to open cervical procedures like reinnervation or thyroplasty as fat resorption and possible continuous muscle atrophy in the case of denervation of the hemi-larynx can lead to decrease of glottic competence over time [31] following fat injection. Our data show a decrease of voice quality parameters over time following significant initial improvement in UVFP/Presbyphonia cases, thus supporting the thesis of progressive fat resorption over time, rather than fat being a permanent augmentation material.

As OM and 3Dex groups were mismatched by a factor of 2, we carefully considered potential sources of bias. To avoid observer and measurement bias, voice specialists conducting follow up were unaware of patient being in 3Dex or OM group and applied the same tests to all patients. Mean patient age was comparable (Table 1) in both groups limiting confounding variables, and all were recruited from the same group of patients with glottic insufficiency from our laryngology consultation to avoid sampling bias. Limitations of our results are the limited follow up period of 12 months and data lost to clinical follow up.

## Conclusions

Our results show that the use of a 3D-Exoscope is a feasible alternative to using a conventional microscope in microlaryngoscopic phonosurgery. The relatively small camera system increases instrument maneuverability and ergonomics for the surgeon, the 3D visualization facilities teaching. Vocal fold augmentation with autologous fat is a good option for patients with glottic insufficiency, however fat resorption and the resulting possible necessity of further interventions need to be discussed with patients suffering from glottic insufficiency.

## Data Availability

The data presented in this study are available upon reasonable request from the corresponding author.
